# Formative-reflective scheme for the assessment of tourism destination competitiveness: an analysis of Italian municipalities

**DOI:** 10.1007/s11135-022-01519-1

**Published:** 2022-09-08

**Authors:** Laura Grassini, Alessandro Magrini, Enrico Conti

**Affiliations:** 1 Department of Statistics, Computer Science, Applications, University of Florence, Florence, Italy; 2 Regional Institute for Economic Planning of Tuscany (IRPET), Florence, Italy

**Keywords:** Composite indicators, Model assessment, Partial least squares, Path modelling, Tourism competitiveness

## Abstract

In this article, we propose a formative-reflective scheme for the assessment of Tourism Destination Competitiveness (TDC) based on a combined use of Partial Least Squares-Path Modelling (PLS-PM) and the method recently proposed by Fattore, Pelagatti, and Vittadini (FPV). TDC is conceived as a construct reflecting the tourism performance of a destination, and several determinants are considered, including endowed resources, created resources, and supporting factors. The proposed scheme is applied to a case study on 1575 Italian municipalities for which the Italian National Institute of Statistics released data on tourist flows. Our contribution is innovative for three aspects: (i) the consistency of the formative-reflective scheme for TDC assessment is discussed on a theoretical basis; (ii) an empirical comparison between PLS-PM and the FPV method is performed; (iii) data with higher granularity than most studies on TDC assessment are employed. Our findings highlight that endowed resources are the primary driver of TDC, followed by created resources and supporting factors, and emphasize that the best ranked destinations are big cities with a multifaceted tourism alongside sea and mountain destinations with cultural attractions.

## Introduction

Partial Least Squares-Path Modelling (PLS-PM) has been widely used in tourism studies (Ali et al. 2017; Assaker et al. 2014; do Valle and Assaker 2016; Esposito Vinzi et al. 2010) and, in particular, in the assessment of Tourism Destination Competitiveness (TDC), with several notable empirical applications in the last decade (Mazanec et al. 2007; Mazanec and Ring 2011; Assaker et al. 2014; Magrini and Grassini 2019; Conti et al. 2020). In the operationalization of TDC, PLS-PM appears more appropriate than composite indicators, because it allows the representation of potential cause-effects relationships hidden in the complex definition of TDC (Mazanec et al. 2007; Mendola and Volo 2017). In fact, PLS-PM can express how the potentialities of a tourism destination, i.e., the sources of comparative and competitive advantages (Crouch and Ritchie 1999; Dwyer and Kim 2003) determine TDC, and may provide a sound informational basis for any managerial decision.

Compared to the traditional methodology of Covariance-Based Structural Equation Modelling (CB-SEM), PLS-PM is particularly more adequate in a prediction perspective (i.e., when the interest is on latent variables scores), in the case of secondary data, and when normality cannot be assumed (Hair et al. 2019a), which are all typical features of TDC analysis. Furthermore, PLS-PM is computationally more feasible than CB-SEM when formative constructs are involved (Hoyle 2012; Hair et al. 2019b). Formative constructs are largely employed in statistical models with Latent Variables (LVs) to comprise Manifest Variables (MVs) of different nature (Borsboom et al. 2003; Edwards and Bagozzi 2000; Bollen 2002; Jarvis et al. 2003), in contrast to reflective constructs which are traditionally used to represent a well defined and structured concept. As such, the use of the formative scheme seems appropriate to represent the determinants of TDC. In fact, the typically adopted model in TDC analysis includes several exogenous formative LVs representing the determinants of TDC (created resources, endowed resources, supporting factors, etcetera), and one endogenous LV representing TDC as a reflection of the performance of a destination in terms of tourist arrivals, tourist receipts, etcetera. In this conceptualisation, each exogenous LV is thought as the synthesis of several MVs of different nature. This particular structural specification involving several formative exogenous LVs and one or more reflective endogenous LVs is often called formative-reflective scheme (Diamantopoulos et al. 2008; Fattore et al. 2018). In the formative-reflective scheme, the exogenous LVs play the double role of summarizing their formative blocks and of mediating, through the system of endogenous LVs, the causal relationships linking formative MVs to reflective ones.

Although PLS-PM is often recommended for formative indicators, several noteworthy shortcomings have been emphasized in the literature, such as the lack of an adequate global goodness of fit measure (Rönkkö et al. 2016). Moreover, Fattore et al. (2018) argued that PLS-PM cannot be properly considered consistent with the formative-reflective scheme because it violates the direction of the causal flow. Specifically, in PLS-PM, the endogenous LVs are built based not only on the exogenous ones (and thus, on the exogenous MVs), but also on the endogenous MVs, which are consequences of endogenous LVs. On these grounds, the authors developed a method (here called FPV from their names: Fattore, Pelagatti, Vittadini) to extract LVs consistently with the formative-reflective scheme. An interesting aspect is that the FPV method is based on the explicit optimization of an objective function depending on a tuning parameter $\alpha$ ranging between 0 and 1, which acts as a weight for the importance of formative blocks against reflective ones. The authors argued that their method can be employed as a diagnostic tool for PLS-PM: the fact that the scores of exogenous LVs and R-squared values vary substantially as a function of $\alpha$ may indicate that extracting meaningful LVs and summarizing the exogenous MVs are incompatible goals.

In this article, we propose a formative-reflective scheme for the assessment of TDC based on a combined use of PLS-PM and FPV: the FPV method is proposed as a validation of PLS-PM, but also as a possible alternative methodology. TDC is conceived as a construct reflecting the tourism performance of a destination (e.g. its attractiveness). Our proposal is applied to a case study on 1575 Italian municipalities for which the Italian National Institute of Statistics (ISTAT) released data on tourist flows. Our work is innovative for three aspects: (i) the consistency of the formative-reflective scheme for TDC assessment is discussed on a theoretical basis; (ii) an empirical comparison between PLS-PM and the FPV method is performed (at the best of our knowledge, the application of FPV on real data is novel); (iii) data with higher granularity than most studies on TDC assessment, which are performed at country level, are employed.

This article is structured as follows. In Sect. 2, noteworthy theoretical frameworks and approaches for the measurement of TDC are presented with special reference to the application of PLS-PM. Section 3 includes a theoretical discussion on the appropriateness of the formative-reflective scheme for TDC analysis, introduces PLS-PM and the related recent criticism, and illustrates the FPV method. Section 4 presents the application of PLS-PM and FPV to the case study on Italian municipalities, where the findings of the two methods are compared and discussed in light of TDC theories. Section 5 contains concluding remarks.

## TDC: theoretical frameworks and empirical analysis

The literature on TDC is very extensive, and even more if we consider the link between TDC and sustainability. In summary, the conceptualization of TDC may be viewed as characterized by three main dimensions (Abreu-Novais et al. 2015): (i) purely economic, i.e., prices differentials, productivity, employment and wealth; (ii) attractiveness, i.e., TDC is conceived as the ability “to increase tourism expenditure, to increasingly attract visitors while providing them with satisfying, memorable experiences, and to do so in a profitable way, while enhancing the well-being of destination residents and preserving the natural capital of the destination for future generations” (Ritchie and Crouch 2003, page 2); (iii) sustainability, i.e., conservation of environmental resources and cultural heritage.

The task of defining and operationalizing TDC is concerned with the identification of the numerous determinants (factors) that contribute to the level of a destination’s competitiveness, and that can be categorized as sources of comparative and competitive advantages (Ritchie and Crouch 2003). Recently, Abreu-Novais et al. (2015), by comparing the noteworthy and comprehensive theoretical frameworks by (Ritchie and Crouch 2003; Dwyer and Kim 2003) and (Heath 2003), found the presence of three common macro-factors: inherited or endowed resources (sources of comparative advantages), created resources (sources of competitive advantages), and external factors (including macro and global situations). On an empirical basis, scholars have fully recognized the complexity (in the sense that it is not directly observable) and the multidimensionality of TDC, therefore both objective and subjective data should be involved in the operationalization of TDC. Two main streams of empirical studies can be distinguished. One stream focuses on specific case studies to identify the key determinants of TDC from subjective data using rigorous methodologies such as the Analytic Hierarchy Process (Lǔstický and Štumpf 2021; Hong 2009), Importance-performance analysis (Azzopardi and Nash 2013; Deng 2007; Enright and Newton 2004), Delphi method (Tanguay et al. 2012). The other stream performs cross-regional analyses based on objective and more comparable (secondary) data making use of composite indicators (Mendola and Volo 2017) or multivariate models representing the cause-effect relationships underlying the concept of TDC (Assaker et al. 2012; Ali et al. 2017).

A variety of studies have recognized that most proposed definitions of TDC involve both its antecedents (i.e., determinants) and outcomes (Mazanec et al. 2007; Mazanec and Ring 2011; Croes 2011; Croes and Kubickova 2013; Assaker et al. 2014), with the former conceived as input variables representing the potentialities of destinations to realize the objectives of tourism development, such as increased demand and enhanced quality of life. In this respect, the use of composite indicators for measuring TDC was often considered not fully appropriate, mainly because they include a mix of both determinants and outcomes of TDC. From a theoretical point of view, Croes and Kubickova (2013) argued that the inclusion of TDC determinants in a composite indicator equates to state that they automatically turn into increased attractiveness and better performance: “Inputs reference the potential of destinations to realize the objectives of tourism development, but potential does not necessarily turn into making a destination more attractive, prompting increased demand and enhanced quality of life” (Croes and Kubickova 2013, page 147). Also, the review in Mendola and Volo (2017) on the use of composite indicators in tourism research reports that authors often mix up output and input elements of TDC with a consequent misinterpretation of the concept. A proposed solution was the development of a performance-based composite indicator of TDC, which is then possibly regressed on TDC determinants Croes (2011); Croes and Kubickova (2013), motivated by the fact that a composite indicator enables a quick global view of a destination’s performance and is able to combine the information on the outcome variables in a robust way. An alternative to composite indicators, and a more appropriate solution for TDC assessment in the view of Mendola and Volo (2017), is the application of multivariate techniques such as structural equation modelling to discover the interrelation between the theoretical dimensions of TDC, and to express how potential cause-effect relationships are transformed into ability. In this regard, the noteworthy contribution by Mazanec et al. (2007) and Mazanec and Ring (2011) paved the way for the structural equations approach with the formative-reflective scheme, and specifically for the use of PLS-PM.

## Formative-reflective scheme for TDC assessment

In this section, we present our formative-reflective scheme for TDC assessment. A justification of the formative-reflective scheme is discussed in Sect. 3.1. Afterwards, PLS-SEM and its traditional validation process is presented in Sect. 3.2, together with the related recent criticism. Finally, the FPV method is detailed in Sect. 3.3 and proposed as a validation of PLS-PM, but also as an alternative estimation method.

### The formative-reflective scheme

The formative-reflective scheme has been widely applied in social and behavioural sciences (Bollen and Bauldry 2011; Bollen and Diamantopulos 2015; Hardin 2017), although an open debate is still in place on how the appropriateness of formative rather than reflective constructs should be established (see, for example, Crocetta et al. 2021; Edwards and Bagozzi 2000; Jarvis et al. 2003; Simonetto 2012). In this respect, an important concern is whether a formative scheme should be interpreted as a set of causal indicators affecting the adjacent LVs or, rather, a composite defined as a linear combination of MVs without a conceptual unity and with weights empirically estimated (Bollen and Bauldry 2011). Often this distinction remains on a theoretical basis because not all the estimation procedures can distinguish between causal and composite indicators, like it is the case of PLS-PM (Bollen and Diamantopulos 2015). In the following, we assume that the formative part of the model consists of indicators expressing the determinants of TDC (i.e., the exogenous constructs). In this case, these indicators may be conceived as the composites that best predict the dependent variable TDC (Edwards and Bagozzi 2000; Heise 1972).

The theoretical framework for the definition of the TDC construct provides a guide in the identification of determinants and outcomes of competitiveness, and may justify the formative-reflective scheme. The indicators of TDC determinants, even though they may be correlated, cannot be considered interchangeable, as they generally do not share a common concept, and they do not have the same antecedents and consequences (Diamantopoulos and Winklhofer 2001; Jarvis et al. 2003; Maggino and Zumbo 2012). More specifically, as discussed above, TDC determinants call in question the sources of comparative and competitive advantages which are proxied by a complex set of indicators relating multifaceted features of a destination. All of these arguments may justify the use of the formative approach (Jarvis et al. 2003, page 3) even because a change in a MV (e.g., the number of natural attractors) does not necessarily imply a change in other MVs (e.g., the number of heritage/cultural attractors). On the other hand, adopting a performance-based measure of TDC (i.e., an endogenous construct), the MVs may be the traditional tourism indicators like tourist flows (arrivals and nights spent) and tourist expenditures or receipts, like in Mazanec et al. (2007), Mazanec and Ring (2011), Assaker et al. (2014), Magrini and Grassini (2019), Conti et al. (2020). In this case, the formative-reflective scheme may represent the cause-effect relationships hidden in the definition of TDC.

### PLS-PM for the formative-reflective scheme

Here we provide a definition of the formative-reflective scheme. Let ${\mathbf {x}}_i=(x_{i,1},\dots ,x_{i,h_i})$ be the *i*-th block of exogenous MVs ($i=1,\ldots ,p$), and ${\mathbf {y}}_j=(y_{j,1},\dots ,y_{j,k_j})$ be the *j*-th block of endogenous MVs ($j=1,\ldots ,q$). Without loss of generality, all the MVs are assumed to have null mean and unit standard deviation. The formative part is defined as:

1 $$\begin{aligned} \xi _i=\varvec{\omega }_i' {\mathbf {x}}_i \quad\quad i=1,\ldots ,p \end{aligned}$$

where $\varvec{\omega }_i$ is a *p*-dimensional vector containing the weights associated to $\xi _i$, one for each MV in ${\mathbf {x}}_i$. The reflective part is defined as:

2 $$\begin{aligned} {\mathbf {y}}_j=\varvec{\lambda }_j \eta _j+\varepsilon _j \quad\quad j=1,\ldots ,q \end{aligned}$$

where $\varvec{\lambda }_j$ is a *q*-dimensional vector containing the loadings for block ${\mathbf {y}}_j$, which represent the correlations between $\eta _j$ and each MV in ${\mathbf {y}}_j$. Denoting $\varvec{\xi }$ as the *p*-dimensional vector of the stacked exogenous LVs and $\varvec{\eta }$ as the *q*-dimensional vector of the stacked endogenous LVs, the structural part of the formative-reflective scheme is expressed as:

3 $$\begin{aligned} \varvec{\eta }={\varvec{\Gamma }}\varvec{\xi } \end{aligned}$$

where ${\varvec{\Gamma }}$ is a $q \times p$ matrix containing the path coefficients, i.e., the coefficients of the system of linear regressions linking each endogenous LV to the exogenous LVs. See Fattore et al. (2018, Fig. 1) for a graphical representation of the model.

PLS-PM estimation of the formative-reflective scheme is based on the PLS algorithm. The PLS algorithm produces two alternative estimates of the LVs’ scores: one as a linear combination of the MVs in its blocks (outer estimation), and the other one as a regression from its predecessors in the structural part (inner estimation). See Esposito Vinzi et al. (2010, pages 49-56) for details. Therefore, the scores of endogenous LVs $\varvec{\eta }$ can be derived from both the reflective and the structural part, i.e., exogenous LVs are explained from both endogenous and exogenous MVs, respectively. Therefore, as far as TDC assessment is concerned, PLS-PM produces a performance-based indicator of TDC, with parameters ${\varvec{\Gamma }}$ of the structural (inner) model expressing how the abilities of a destination turn into performance.

Since PLS-PM does not have a global optimization criterion, the validity of the extracted LVs is checked by means of several diagnostics depending on the specification of the constructs, i.e., formative or reflective. For reflective constructs, each MV should have a loading no less than 0.5 and, ideally, greater than 0.7 (Hair et al. 2010). This requirement is motivated by the fact that loadings equal to 0.5 and 0.75 indicate, respectively, that the reflective LV explains 25% and 50% of the variance of the MV. Afterwards, the check of convergent validity, discriminating validity and composite reliability is typically carried out. The convergent validity of a reflective construct is assessed by inspecting the Average Variance Extracted (AVE): it represents the average proportion of variance of the MVs in the block explained by the LV. The commonly recommended minimal threshold of the AVE is 0.5 (Bagozzi and Yi 1988; Henseler et al. 2009; Hair et al. 2014), implying that the construct explains at least half the variance of the MVs in its block. The discriminant validity of a reflective construct is typically checked in three ways: (i) the AVE must be higher than the squared correlations between the LV and the other LVs (Fornell and Larcker 1981); (ii) for each MV in the block, the loading must be higher than the correlation with the other LVs (Farrell 2010); (iii) if there are more than one reflective construct, the heterotrait-monotrait (HTMT) ratios must be lower than 0.85 (Henseler et al. 2015). Composite reliability of a reflective construct is checked by inspecting the composite reliability index, which should not be lower than 0.7 (Nunnally 1978).

For formative constructs, the commonly adopted diagnostics require to check: (i) multicollinearity through Variance Inflation Factors (VIFs), (ii) positivity and statistical significance of the weights, (iii) the R-squared value. Multicollinearity check is motivated by the fact that MVs in a formative block should not be highly correlated. Commonly adopted maximal thresholds for VIFs of the MVs in a formative block are 3.33 (Diamantopoulos and Siguaw 2006) and 10 (Mathieson et al. 2001). Positive sign and significance of all the weights of MVs in a formative block is required to interpret the formative LV as a composite indicator: on one hand, negative weights are senseless and, on the other hand, a MV with weight of negligible magnitude does not effectively contribute to the composite. The R-squared of a formative construct is similar to the AVE of a reflective LV, but it provides a different information: a formative LV is caused by the MVs in its block, thus the R-squared represents the proportion of variance of the formative construct explained by the MVs. The commonly recommended minimal threshold for the R-squared in empirical applications is 0.5 (Edwards 2001; MacKenzie et al. 2011), implying that the MVs in a formative block explain at least half the variance of the formative construct.

Recently, several criticism has been moved to PLS-PM, especially about the estimation of the reflective part. In particular, it has been emphasized the absence of a global optimization criterion, making the estimation procedure articulated and heuristic, and forcing to use empirical confidence intervals from the bootstrap distribution in order to test hypotheses on parameters (Rönkkö and Evermann 2013; Rönkkö et al. 2016). Also, Fattore et al. (2018) recently emphasized the violation of the causal flow of the PLS algorithm, noting that the endogenous LVs are build based not only on the exogenous ones (and thus, on the exogenous MVs), but also on the endogenous MVs, which are consequences of endogenous LVs. As such, the traditional validation methodology for PLS-PM is not properly suited to assess the validity of the formative-reflective scheme. In this context, the method proposed by Fattore et al. (2018), that we call FPV from the names of the authors (Fattore, Pelagatti, Vittadini), may be a valuable integration of the traditional validation methodology for PLS-PM, and even an alternative estimation method overcoming the lack of a global optimization criterion.

### The FPV method

The FPV method (Fattore et al. 2018) defines two loss functions, one for the formative part (*x*-side loss function), and the other one for the reflective part (*y*-side loss function). The *x*-side loss function considers how well, in each formative block, each MV predicts the exogenous LV:

4 $$\begin{aligned} L_x(\varPi , {\varvec{\Omega }})= \frac{1}{p} \sum _{i=1}^{p} \frac{{\mathbb {T}}\text {r} \{{\mathbb {E}}[({\mathbf {x}}_i-\varvec{\pi }_i \varvec{\omega }_i' {\mathbf {x}}_i)({\mathbf {x}}_i-\varvec{\pi }_i \varvec{\omega }_i' {\mathbf {x}}_i)'] \}}{{\mathbb {T}}\text {r}\{{\mathbb {E}}[{\mathbf {x}}_i {\mathbf {x}}_i']\}} \end{aligned}$$

where $\varvec{\pi }_i$ is a vector of $h_i$ regression coefficients, $\varPi$ is the matrix obtained by stacking the vectors $\varvec{\pi }_i$ ($i=1,\ldots ,p$) by row, and ${\varvec{\Omega }}$ is the matrix obtained by stacking the vectors $\varvec{\omega }_i$ ($i=1,\ldots ,p$) by row. Note that this loss function is minimized when $\varvec{\omega }_i$ is the first eigenvector of ${\mathbb {E}}[{\mathbf {x}}_i {\mathbf {x}}_i']$, so that $\xi_i$ is the first principal component of $\mathbf{x}_i$. The *y*-side loss function considers how well each endogenous LV predicts each MV in its block:

5 $$\begin{aligned} L_y({\varvec{\Omega }},{\varvec{\Gamma }},{\varvec{\Lambda }})= \frac{1}{q} \sum _{j=1}^{q} \frac{{\mathbb {T}}\text {r} \{{\mathbb {E}}[({\mathbf {y}}_j-\varvec{\lambda }_j \varvec{\gamma }_j' {\varvec{\Omega }} {\mathbf {x}}) ({\mathbf {y}}_j-\varvec{\lambda }_j \varvec{\gamma }_j' {\varvec{\Omega }} {\mathbf {x}})'] \}}{{\mathbb {T}}\text {r}\{{\mathbb {E}}[{\mathbf {y}}_j {\mathbf {y}}_j']\}} \end{aligned}$$

where $\varvec{\lambda }_j$ is a vector of $k_j$ regression coefficients, ${\varvec{\Lambda }}$ is the matrix obtained by stacking the vectors $\varvec{\lambda }_j$ ($j=1,\ldots ,q$) by row, and $\varvec{\gamma }_j$ is the *j*-th row of the matrix of path coefficients ${\varvec{\Gamma }}$. Note that $L_y$ can be interpreted as the average of normalized residual variances.

The global loss function is defined by balancing $L_x$ and $L_y$ through a tuning parameter $0 \le \alpha \le 1$:

6 $$\begin{aligned} L^{(\alpha )}({\varvec{\Pi }}, {\varvec{\Omega }}, {\varvec{\Gamma }},{\varvec{\Lambda }})=(1-\alpha ) L_x({\varvec{\Pi }},{\varvec{\Omega }}) +\alpha L_y({\varvec{\Omega }},{\varvec{\Gamma }},{\varvec{\Lambda }}) \end{aligned}$$

When $\alpha =0$, each exogenous LV is the first principal components of its block of MVs. In this case, the extracted variance of the formative constructs is maximized. When $\alpha =1$, the global loss function reduces to the minimization of $L_y$, thus the exogenous LVs are linear combinations of the respective blocks of MVs that best predict, through the endogenous LVs, the endogenous MVs. In this case, we have a multivariate regression problem with a complex system of constraints, implied by the particular form of the matrix of path coefficients ${\varvec{\Gamma }}$. Note that $R^2_x= 1-L_x$ is the mean of the *p* R-squared values for the *x*-side, and $R^2_y= 1-L_y$ is the mean of the *q* R-squared values for the *y*-side.

The minimization of the global loss function is made numerically for given values of $\alpha$. For this reason, the authors argued that their procedure can be considered as a PLS-PM soft modelling approach. Moreover, they state that, if the model is well designed, the choice of $\alpha$ is irrelevant, i.e., the scores of exogenous LVs and both $R^2_x$ and $R^2_y$ should vary little with $\alpha$. For this reason, they suggested two diagnostics: (i) for each exogenous LV $\xi _i$, the correlation between $\xi _i$ given any value of $\alpha$ and $\xi _i$ given $\alpha =0$, called *rotation* and denoted by $\rho _{\xi _i}$; (ii) the values of $R^2_x$ and $R^2_y$ as a function of $\alpha$. In the view of the authors, the fact that these features change as a function of $\alpha$ is a symptom that extracting meaningful LVs and summarizing the exogenous MVs are incompatible goals and, as a consequence, the formative-reflective scheme is not appropriate. At the purpose of assessing the consistency of PLS-PM with the formative-reflective scheme, we extend the two diagnostics above: the first to exogenous LVs extracted by PLS-PM, and the second to $R^2_x$ and $R^2_y$ of PLS-PM estimation. In PLS-PM, $R^2_x$ corresponds to the mean squared correlations between each exogenous LV and the MVs in its block, while $R^2_y$ equates to the mean redundancy of endogenous blocks. However, as shown by several examples on simulated data in Fattore et al. (2018, Sect. 4), the value of $\alpha$ making FPV estimates similar to PLS-PM ones depends on the correlation structure of the data, thus, in general, some PLS-PM parameters may be more similar to FPV ones in the case $\alpha =0$ than in the case $\alpha =1$.

Regarding the estimation procedure of the FPV method, two interesting points should be discussed: one related to the formative (exogenous) and the other to the reflective (endogenous) part of the model. In introducing their approach, the authors of the FPV method claimed the fact that their algorithm allows a good fit of the exogenous MVs. However, the aim of a formative measure is not necessarily the best representation of the covariation of its MVs. The use of PCA, even though proposed by some scholars to identify redundant formative indicators (Bollen 2002; Nardo et al. 2005), has been recently criticized in Mazziotta and Pareto (2019), who observed that PCA risks to neglect less correlated MVs although they are important conceptually. However, the role of PCA in the FPV method is regulated by the tuning parameter $\alpha$ and, in particular, PCA impacts less as $\alpha$ increases, being neglected when $\alpha =1$, which is the case logically closest to PLS-PM. The other concern on the FPV method is about the endogenous LVs that are completely determined by the exogenous LVs. Differently from PLS-PM (and also from CB-SEM), the FPV method does not produce automatically a LV from the reflective measurement model. Therefore, it is apparent that the FPV method does not involve a measurement model for the endogenous reflective constructs. Thus, in the perspective of TDC assessment, the logic underlying the FPV method is not to build a performance-based measure of TDC but, rather, TDC comes to express the potentialities of the destination that can be turn into TDC outcomes represented by the endogenous MVs.

## Application to Italian municipalities

The empirical analysis focuses on Italian municipalities and is based on data from the Italian National Statistical Institute (ISTAT) for 2015. Although the data is a bit outdated, they allow a comparison with recent empirical analysis on Italian municipalities (Conti et al. 2020). Our dataset consists of 1575 municipalities out of 7903, which are those for which ISTAT released data on tourist flows in 2015. Despite this reduction in the number of units of analysis, the coverage of the dataset in terms of tourist flows is satisfactory: the percentage of overnight stays covered by the data is: 92.6% for the whole Italy and from 78.0% to 97.4% across the 20 Italian regions (see Table 1). In Sect. 4.1, we describe the units of analysis, while latent constructs and indicators comprised in the model are detailed in Sect. 4.2.

**Table 1:** Coverage of the sample with respect to overnight stays across the 20 Italian regions. ‘Sample’: sum of overnight stays by region within the sample. ‘Actual’: total overnight stays by region

Region	Sample	Actual	Coverage (%)
Abruzzo	5,736,103	6,177,230	92.9
Basilicata	2,089,778	2,302,678	90.8
Calabria	6,790,263	8,151,234	83.3
Campania	17,411,483	18,855,907	92.3
Emilia-Romagna	35,616,604	36,561,539	97.4
Friuli-Venezia Giulia	7,462,247	7,915,817	94.3
Lazio	30,854,537	31,679,914	97.4
Liguria	13,287,286	14,328,278	92.7
Lombardia	29,510,583	37,857,240	78.0
Marche	11,036,172	12,144,715	90.9
Molise	393,154	492,018	79.9
Piemonte	10,823,296	13,681,316	79.1
Puglia	12,729,694	13,526,151	94.1
Sardegna	11,795,764	12,392,827	95.2
Sicilia	13,377,790	14,510,708	92.2
Toscana	42,649,098	44,379,574	96.1
Trentino-Alto Adige	43,237,326	45,510,559	95.0
Umbria	5,428,852	5,910,632	91.8
Valle d’Aosta	2,924,509	3,238,559	90.3
Veneto	60,515,743	63,257,174	95.7
Italy (total)	363,670,282	392,874,070	92.6

### Units of analysis

In tourism studies, it is possible to find different definitions of tourist destination (Capone and Boix 2008). In particular, although recognizing that a tourist destination can also be a perceptual concept, Buhalis (2000, p. 98) stated that it is “a defined geographic region that is understood by its visitors as a single entity, with a political and legislative framework for marketing and tourism planning”. Furthermore, according to WTO (2004, p. 8) a tourist destination “has physical and administrative boundaries defining its management, and images and perceptions defining its market competitiveness. Local destinations incorporate various stakeholders often including a host community and can nest and network to form larger destinations”.

When a destination management organization responsible for strategically planning the development of tourist areas does exist, it usually involves local authorities, mainly because the local government has a decisive role in controlling and supporting landscape, historical resources and public assets. Therefore, a tourist destination can be any territorial unit with specific characteristics and administrative responsibility, thus the municipality level of analysis seems appropriate. Furthermore, the more local the level, the more an administrative entity takes into account the issues of economic, social and environmental sustainability. The fact that the municipality level is a sound territorial area for tourism studies is also proved by those contributions concerning the implementation of systems of tourism indicators for competitiveness and sustainability (Torres-Delgado and Palomeque 2014).

At the best of our knowledge, only Alves and Nogueira (2015) and Conti et al. (2020) have analysed TDC on municipality data using structural equation models. Alves and Nogueira (2015) applied PLS-PM for studying the competitiveness of Brazilian municipalities and found the predominance role of the ‘Tourism Infrastructure’ construct (i.e., sources of competitive advantages), followed by ‘Heritage and Culture’ (i.e., sources of comparative advantages). Similar results were obtained in Conti et al. (2020), who applied PLS-PM on data covering all Italian municipalities. In Conti et al. (2020), differently from the present article, many missing data were imputed because the information on tourist flows was not available for all municipalities.

### Constructs and indicators

**Fig. 1:**
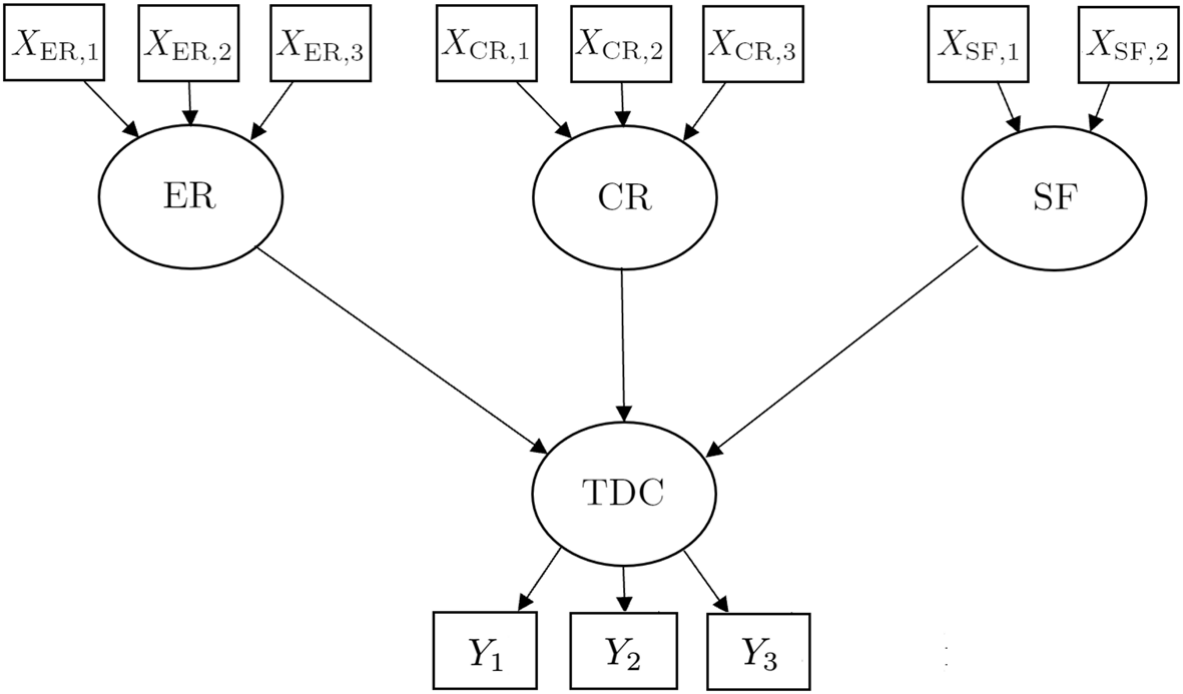
Path diagram of the model for our case study in TDC assessment of Italian municipalities

Our model for TDC assessment of Italian municipalities consists of one endogenous construct (TDC) and three exogenous constructs (TDC determinants), i.e., Endowed Resources (ER), Created Resources (CR), and Supporting Factors (SF), for a total of eleven indicators. The path diagram of the model is displayed in Fig. 1, while source and summaries of the data are shown in Table 2. In the following, the constructs and their indicators are described. Note that we also postulated a fourth exogenous construct, Sustainability (SUS), but it was removed because the weights of its indicators were not significant (see Sect. 4.2.2 and Table 2).

**Table 2:** Source and data summaries for the selected indicators. ISTAT: Italian Statistical Institute; ISPRA: Higher Institute for Environmental Protection and Research; ASIA: statistical register of active Italian firms; OMI: quotation database managed by the Italian Revenue Agency; MSE: Italian ministry of economic development; DB Appalti: database of Italian public procurements

Indicator	Measurement	Source	Mean	Std. dev.
$Y_1$	Geometric mean: number of overnight stays to resident population (stays/person) $\times$ number of overnight stays to surface area (stays/squared km).	ISTAT	0.934	1.994
$Y_2$	Percentage of occupied beds out of total beds.	ISTAT	19.7	11.6
$Y_3$	Average market value of dwelling houses (Euro/squared meters).	OMI	1645.2	984.3
$X_{\text {ER},1a}$	Coastline length to surface area (km/squared km).	ISTAT	129.2	413.0
$X_{\text {ER},1b}$	Percentage of area above 1500 meters out of total surface area.	ISPRA	11.5	25.3
$X_{\text {ER},1}$	Holiday sites: composite of $X_{\text {ER},1a}$ and $X_{\text {ER},1b}$.	Our computation	0.000	0.655
$X_{\text {ER},2}$	Number of museums to surface area (museums/squared km).	ISTAT	0.033	0.076
$X_{\text {ER},3}$	Dichotomous: 1 if at least one Unesco World Heritage site, 0 otherwise.	Unesco	0.114	0.317
$X_{\text {CR},1}$	Number of restaurants with Michelin stars to surface area (restaurants/squared km).	Michelin	0.005	0.031
$X_{\text {CR},2}$	Geometric mean: number of bed places to resident population (beds/person) $\times$ number of bed places to surface area (beds/squared km).	ISTAT	0.107	0.060
$X_{\text {CR},3}$	Percentage of employees in tourism services out of total employees.	ASIA	14.4	26.2
$X_{\text {SF},1}$	Percentage of resident population served by fixed or mobile broadband.	MSE	88.8	21.1
$X_{\text {SF},2}$	Investments in communication routes to surface area (thousand Euro/squared kilometres).	DB Appalti	272.0	943.0
$X_{\text {SUS},1}$	% waste in separate collection	ISPRA	49.11	22.1
$X_{\text {SUS},2}$	Protected natural parks (0/1 dummy)	Comuniverso	0.230	0.421

#### Endogenous construct

As anticipated in Sect. 2, we adopt a performance-based measure of TDC describing the level of tourism development. At this purpose, we selected the following three indicators:

- *overnight stays* ($Y_1$), measured as the geometric mean between number of overnight stays to resident population (stays/person) and the number of overnight stays to surface area (stays/squared km);
- *gross occupancy rate* ($Y_2$), measured as the percentage of occupied beds out of total beds, disregarding whether the accommodation facilities are actually open or not;
- *real estate value* ($Y_3$), measured as the average market value of dwelling houses (Euro/squared meters).

While overnight stays and the gross occupancy rate are the two typical indicators of a destination’s performance based on tourism flows, the real estate value is exploited as a proxy of the effects of the hidden economy of tourism and of all accommodation facilities that are not captured by current official statistics on tourism flows.

We recognize that this specification is far from a comprehensive vision of TDC which, according to the theoretical definition, should also include well-being measures. However, well-being indicators focused on tourism mostly consist of subjective data and are not available at a local territorial scale.

#### Exogenous constructs

The variables selected to express the determinants of TDC aim at reflecting the so called comparative and competitive advantages of a destinations, where “comparative advantages constitute the resources available to a destination, competitive advantages relate to a destination’s ability to use these resources effectively over the long-term” (Crouch and Ritchie 1999, p. 143). The model proposed by Dwyer and Kim (2003) is the reference framework for identifying the determinants of TDC and the corresponding observable indicators (Cvelbar et al. 2016). Based on the Dwyer & Kim framework, we defined three exogenous LVs expressing:

1. endowed (inherited) resources (labelled as ER), that can be natural, heritage, or cultural, and are potential sources of comparative advantages;
2. created resources (labelled as CR), which are concerned with the equipment that a destination requires in order to sustain tourist demand, and include tourism infrastructure and organization;
3. supporting factors (labelled as SF), which provide the conditions required to establish a successful tourism industry (e.g., communication infrastructures), even though they are not specifically designed for the tourism industry.

The construct Endowed Resources (ER) aims at representing the equipment of attractions received as inheritance by a destination: natural beauty, landscape, historical and artistic heritage. The measurement of this LV is a difficult task, because subjective aspects are also involved. We considered three indicators for the ER construct:

i) *holiday sites* ($X_{\text {ER,1}}$), combining coastal ($X_{\text {ER,1a}}$) and mountain ($X_{\text {ER,1b}}$) natural resources. In order to account for the fact that most destinations have more than one type of natural resources (i.e., both coastal and mountain), we merged the indicators $X_{\text {ER,1a}}$ and $X_{\text {ER,1b}}$ into a unique one: the two indicators were standardized subtracting the mean and dividing by the standard deviation, then their average was computed. The standardization is required as the two variables have different unit of measurement;
ii) *museums* ($X_{\text {ER,2}}$), measured as number of museums to surface area (museums/squared km);
iii) *world heritage sites* ($X_{\text {ER,3}}$), expressed as a dichotomous variable taking value 1 in case of presence of at least one Unesco World Heritage Site, and value 0 otherwise.

The construct Created Resources (CR) was conceived to represent the equipment that a destination requires in order to sustain continuous tourist demand. Specifically, we employed the following three indicators:

i) *high quality restaurants* ($X_{\text {CR,1}}$), quantified by the number of restaurants with Michelin stars to surface area (restaurants/squared km);
ii) *bed places* ($X_{\text {CR,2}}$), measured as the geometric mean between number of bed places to resident population (beds/person) and number of bed places to surface area (beds/squared km);
iii) *tourism employment* ($X_{\text {CR,3}}$), measured as the percentage of employees in tourism services out of total employees.

The construct Supporting Factors (SF) describes the infrastructures supporting the supply of tourism services. For this construct, we employed the following two indicators:

- *broadband coverage* ($X_{\text {SF,1}}$), measured as the percentage of resident population served by fixed or mobile broadband;
- *quality of communication routes* ($X_{\text {SF,2}}$), measured as investments in communication routes to surface area (thousand Euro/squared kilometres).

We also postulated a fourth determinant of TDC, Sustainability (SUS), measured through two indicators: the percentage of waste in separate collection ($X_{\text {SUS},1}$), and the absence/presence of protected areas ($X_{\text {SUS},2}$). Descriptive statistics of these two indicators are reported in Table 2 but, since PLS weights were not significant, the construct SUS was removed from the model. Note that the number of indicators in a formative construct has strong implications for the statistical significance and the magnitude of weights, even when the indicators are weakly correlated (Cenfetelli and Bassellier 2009). For this reason, we paid attention to maintain a balance in the number of indicators across the LVs.

### Results

In this section, we present the results of the application of our formative-reflective scheme to the case study on Italian municipalities. PLS-PM and FPV estimation were performed using the R packages plspm (Sanchez et al. 2015) and pathmod (Pelagatti 2020), respectively.

Given the different scales of measure, the indicators have been preliminary standardized subtracting the mean and dividing by the standard deviation.

#### PLS-PM results

Table 3 reports the results of PLS-PM estimation, specifically the weights of formative indicators, the loadings of TDC indicators and the path coefficients describing the relationship between TDC and its determinants. In the table, bias-corrected 95% confidence intervals based on 5000 bootstrap resamples are shown within brackets. All intervals do not contain value 0, thus we conclude that all weights, loadings and path coefficients are statistically significant at 5% level.

**Table 3:** Weights, loadings and path coefficients resulting from PLS-PM estimation. Bias-corrected 95% confidence intervals based on 5000 bootstrap resamples are shown within brackets

Formative part	
Weights	Estimate
$X_{\text {ER},1}\longrightarrow \text {ER}$	0.659 (0.542, 0.763)
$X_{\text {ER},2}\longrightarrow \text {ER}$	0.514 (0.341, 0.681)
$X_{\text {ER},3}\longrightarrow \text {ER}$	0.333 (0.187, 0.464)
$X_{\text {CR},1}\longrightarrow \text {CR}$	0.759 (0.564, 0.866)
$X_{\text {CR},2}\longrightarrow \text {CR}$	0.361 (0.226, 0.522)
$X_{\text {CR},3}\longrightarrow \text {CR}$	0.481 (0.318, 0.659)
$X_{\text {SF},1}\longrightarrow \text {SF}$	0.601 (0.438, 0.767)
$X_{\text {SF},2}\longrightarrow \text {SF}$	0.733 (0.566, 0.848)

Reflective part	
Loadings	Estimate
$\text {TDC}\longrightarrow Y_{1}$	0.708 (0.643, 0.770)
$\text {TDC}\longrightarrow Y_{2}$	0.696 (0.646, 0.744)
$\text {TDC}\longrightarrow Y_{3}$	0.887 (0.859, 0.907)

Structural part	
Path coefficients	Estimate
$\text {ER}\longrightarrow \text {TDC}$	0.324 (0.276, 0.386)
$\text {CR}\longrightarrow \text {TDC}$	0.272 (0.214, 0.333)
$\text {SF}\longrightarrow \text {TDC}$	0.249 (0.198, 0.295)

For what concerns the formative part, the estimated weights are significantly greater than 0, indicating that each exogenous block, representing a summary of each considered TDC determinant, can be meaningfully interpreted as a composite indicator. Furthermore, the weights can be compared each other because data have been standardized. Specifically, the indicator most contributing to define the composite of endowed resources (ER) is holiday sites (weight: 0.659), followed by museums (weight: 0.514) and Unesco sites (weight: 0.333). The indicator most contributing to define the composite of created resources (CR) is high quality restaurants (weight: 0.759), followed by employment in tourism services (weight: 0.481) and bed places (weight: 0.361). Finally, the indicator most contributing to define the composite of supporting factors (SF) is investments in communication routes (weight: 0.733), followed by broadband coverage (weight: 0.601).

For what concerns the reflective part, we see that the average market value of dwelling houses ($X_{\text {TDC},3}$) is the indicator with the highest correlation with the TDC construct (loading: 0.887), followed by overnight stays ($X_{\text {TDC},1}$) and gross occupancy rate ($X_{\text {TDC},2}$), which show a similar correlation with TDC (0.708 and 0.696, respectively).

For what concerns the structural part, the endowed resources (ER) construct has the highest path coefficient (0.324), thus it results the most important TDC determinant, followed by created resources (CR, path coefficient: 0.272) and supporting factors (SF, path coefficient: 0.249).

The traditional diagnostics to validate the LVs extracted by PLS-PM estimation are provided in Table 4. For the reflective construct TDC, all loadings are near or above 0.7 (see Table 3) suggesting the validity of the endogenous indicators, the composite reliability index greater than 0.7 indicates good composite reliability, and the AVE equal to 0.59 denotes good convergent validity. Also, the AVE of the TDC construct is greater than its squared correlations with the other constructs, indicating adequate discriminant validity. As a further check of the adequacy of the TDC construct, we inspected the first eigenvalue of the correlation matrix of endogenous indicators, which resulted greater than 1. For formative constructs (TDC determinants), all weights are significant and positive (see Table 3) making meaningful to interpret exogenous LVs as composite indicators, VIFs are all near the minimum value 1 thus highlighting no problem of multicollinearity, and the R-squared values are all above the threshold 0.25. In summary, traditional diagnostics suggest an adequate specification for both reflective and formative constructs.

**Table 4:** Diagnostic indices for PLS-PM estimation. CRI: convergent reliability index; AVE: average variance extracted; VIF: variance inflation factor; ‘1st eigen.’: first eigenvalue of the correlation matrix of indicators

Formative part			
Construct	$\text {R}^2$	Indicator	VIF
ER	0.414	$X_{\text {ER},1}$	1.031
		$X_{\text {ER},2}$	1.033
		$X_{\text {ER},3}$	1.027
CR	0.356	$X_{\text {CR},1}$	1.007
		$X_{\text {CR},2}$	1.002
		$X_{\text {CR},3}$	1.008
SF	0.557	$X_{\text {SF},1}$	1.013
		$X_{\text {SF},2}$	1.013

Reflective part			
Construct	Index		Value
TDC	CRI		0.810
	AVE		0.590
	1st eigen.		1.789

Squared correlations			
	ER	CR	SF
CR	0.085		
SF	0.007	0.004	
TDC	0.180	0.123	0.067

#### FPV results and comparison with PLS-PM

Table 5 reports weights (for formative MVs), loadings (for reflective MVs) and path coefficients estimated by PLS-PM and by FPV as a function of $\alpha$. For a proper comparison with FPV, we show the correlations between TDC scores predicted by the structural part of PLS-PM (called ‘fitted TDC’ from now on) and endogenous MVs, instead of the usual PLS-PM loadings, which are shown in Table 3. We see that parameters that change most as a function of $\alpha$ are the weights of $X_{\text {ER},1}$ ($-0.246$), $X_{\text {CR},2}$ (−0.533) and $X_{\text {CR},3}$ ($+0.306$), and the path coefficient of ER on TDC (−0.215). From what concerns the difference between PLS-PM and FPV estimation, we see that some PLS-PM estimates are more similar to FPV ones in the case $\alpha=0$ than in the case $\alpha=1$, i.e., the weights of $X_{\text {ER},2}$, $X_{\text {CR},2}$, $X_{\text {SF},1}$ and $X_{\text {SF},2}$, and the path coefficient of ER on TDC.

**Table 5:** Weights, loadings and path coefficients resulting from the FPV method

Parameter	$\alpha =0.0$	$\alpha =0.1$	$\alpha =0.2$	$\alpha =0.3$	$\alpha =0.4$	$\alpha =0.5$
*Weights*						
$X_{\text {ER},1}\longrightarrow \text {ER}$	0.520	0.549	0.580	0.610	0.638	0.665
$X_{\text {ER},2}\longrightarrow \text {ER}$	0.527	0.513	0.500	0.486	0.470	0.454
$X_{\text {ER},3}\longrightarrow \text {ER}$	0.498	0.480	0.459	0.438	0.418	0.397
$X_{\text {CR},1}\longrightarrow \text {CR}$	0.624	0.597	0.584	0.582	0.582	0.584
$X_{\text {CR},2}\longrightarrow \text {CR}$	0.212	0.362	0.448	0.504	0.545	0.575
$X_{\text {CR},3}\longrightarrow \text {CR}$	0.699	0.662	0.623	0.583	0.548	0.518
$X_{\text {SF},1}\longrightarrow \text {SF}$	0.670	0.680	0.691	0.702	0.714	0.725
$X_{\text {SF},2}\longrightarrow \text {SF}$	0.670	0.659	0.648	0.636	0.623	0.611
*Loadings*						
$\text {TDC}\longrightarrow X_{\text {TDC},1}$	0.349	0.354	0.354	0.353	0.352	0.352
$\text {TDC}\longrightarrow X_{\text {TDC},2}$	0.250	0.266	0.278	0.286	0.292	0.296
$\text {TDC}\longrightarrow X_{\text {TDC},3}$	0.549	0.556	0.560	0.562	0.564	0.565
*Path coefficients*						
$\text {ER}\longrightarrow \text {TDC}$	0.302	0.326	0.352	0.378	0.403	0.427
$\text {CR}\longrightarrow \text {TDC}$	0.359	0.339	0.323	0.314	0.308	0.304
$\text {SF}\longrightarrow \text {TDC}$	0.241	0.259	0.272	0.284	0.294	0.304

Parameter	$\alpha =0.6$	$\alpha =0.7$	$\alpha =0.8$	$\alpha =0.9$	$\alpha =1.0$	PLS-PM
*Weights*						
$X_{\text {ER},1}\longrightarrow \text {ER}$	0.690	0.713	0.733	0.750	0.766	0.659
$X_{\text {ER},2}\longrightarrow \text {ER}$	0.438	0.422	0.407	0.392	0.379	0.514
$X_{\text {ER},3}\longrightarrow \text {ER}$	0.378	0.360	0.344	0.330	0.317	0.333
$X_{\text {CR},1}\longrightarrow \text {CR}$	0.588	0.593	0.598	0.604	0.609	0.759
$X_{\text {CR},2}\longrightarrow \text {CR}$	0.599	0.619	0.634	0.645	0.655	0.361
$X_{\text {CR},3}\longrightarrow \text {CR}$	0.488	0.461	0.436	0.414	0.394	0.481
$X_{\text {SF},1}\longrightarrow \text {SF}$	0.734	0.743	0.752	0.759	0.766	0.601
$X_{\text {SF},2}\longrightarrow \text {SF}$	0.600	0.589	0.578	0.569	0.560	0.733
$$\textit {Loadings}$$						
$\text {TDC}\longrightarrow X_{\text {TDC},1}$	0.351	0.351	0.350	0.350	0.350	0.334$^{(*)}$
$\text {TDC}\longrightarrow X_{\text {TDC},2}$	0.299	0.301	0.302	0.303	0.304	0.302$^{(*)}$
$\text {TDC}\longrightarrow X_{\text {TDC},3}$	0.566	0.567	0.568	0.568	0.568	0.558$^{(*)}$
Path coefficients						
$\text {ER}\longrightarrow \text {TDC}$	0.449	0.469	0.488	0.503	0.517	0.324
$\text {CR}\longrightarrow \text {TDC}$	0.301	0.300	0.299	0.300	0.300	0.272
$\text {SF}\longrightarrow \text {TDC}$	0.312	0.320	0.327	0.333	0.339	0.249

$^{(*)}$: for a proper comparison with FPV, we show the correlations between TDC scores predicted by the structural part of PLS-PM (‘Fitted TDC’) and endogenous MVs, instead of the usual PLS-PM loadings, which are equal to 0.708, 0.696 and 0.887, respectively (see Table 3)

**Fig. 2:**
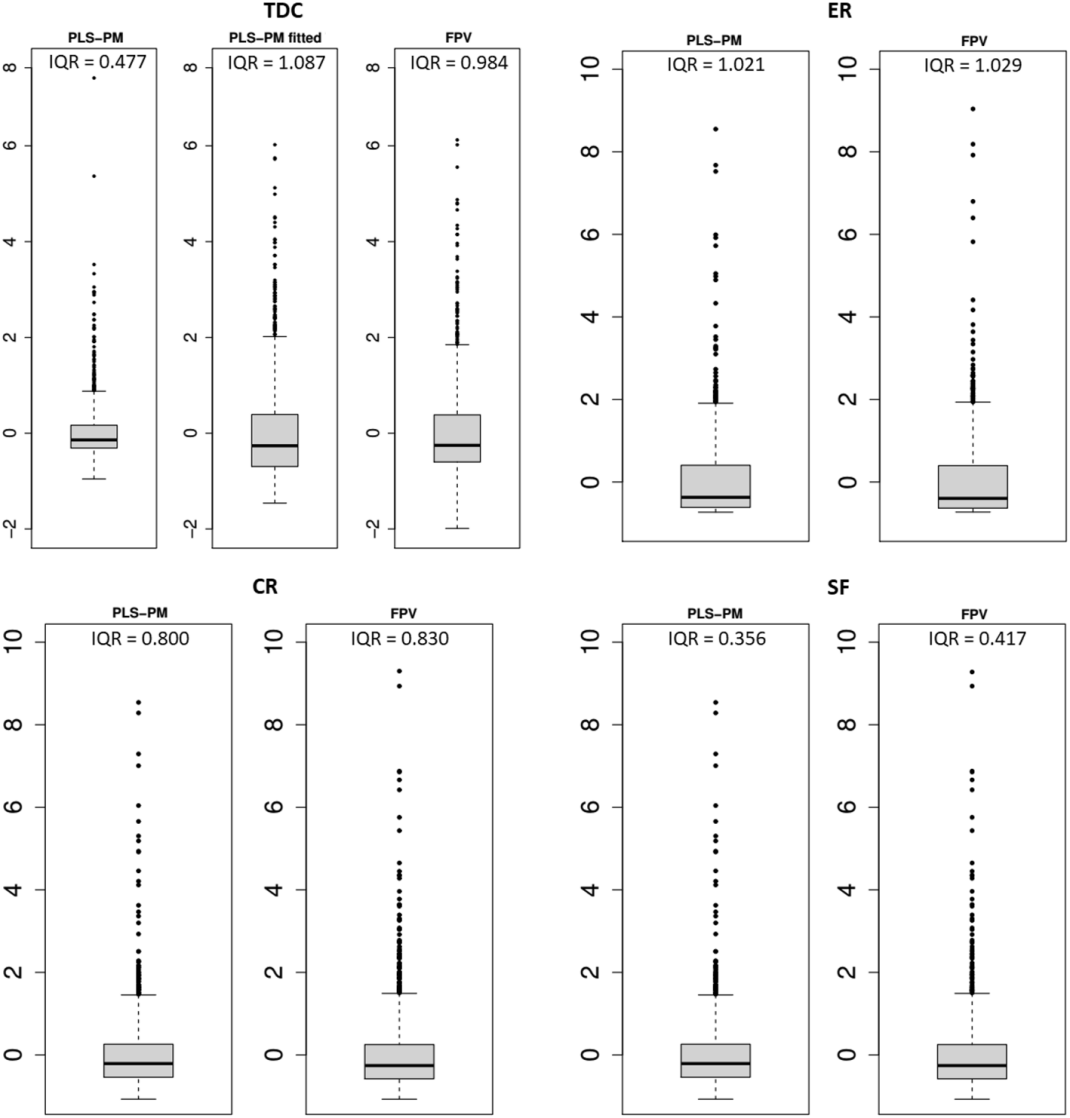
Box plots of scores extracted by PLS-PM and FPV with $\alpha =1$. ‘Fitted TDC’ refers to TDC scores predicted by the structural part of PLS-PM. ‘IQR’: interquartile range

**Fig. 3:**
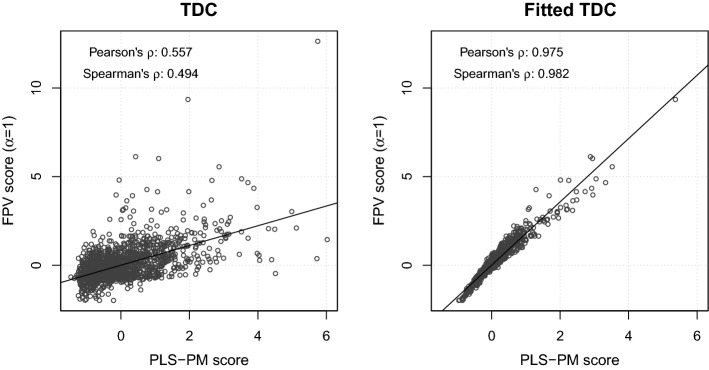
Scatter plots of TDC scores extracted by PLS-PM and by FPV with $\alpha =1$. ‘Fitted TDC’ refers to TDC scores predicted by the structural part of PLS-PM. In each graphic, the regression line is shown, and Pearson’s and Spearman’s correlations are reported

Anyway, if we inspect the box plots of LV scores extracted by PLS-PM and FPV with $\alpha =1$ (Figure 2) and their correlations (Table 6), we find a substantial agreement among the scores of the same construct, excepting for the endogenous construct TDC, for which the scores extracted by FPV appears more similar to those predicted by the structural part of PLS-PM (‘fitted TDC’), rather than to the ones extracted by PLS-PM. This finding clearly emerges also from the scatter plots of TDC scores extracted by the two methods (Fig. 3).

**Table 6:** Correlations between PLS-PM and FPV scores with $\alpha =1$. ‘Fitted TDC’ refers to TDC scores predicted by the structural part of PLS-PM

FPV scores	PLS-PM scores				
$\alpha =1$	ER	CR	SF	Fitted TDC	TDC
ER	0.987	0.300	0.036	0.753	0.419
CR	0.220	0.941	−0.105	0.552	0.330
SF	0.049	−0.069	0.975	0.440	0.252
TDC	0.796	0.636	0.403	0.975	0.557

**Fig. 4:**
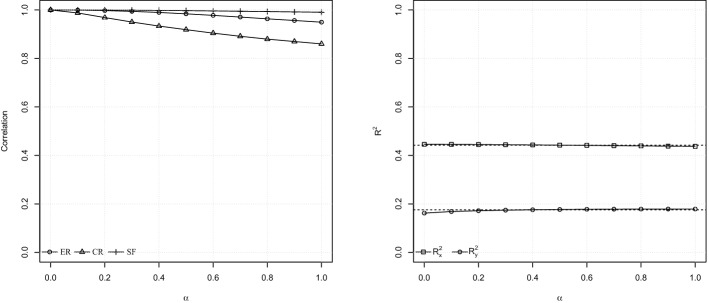
FPV diagnostics. Left panel: rotation of exogenous LVs. Right panel: *x*-side and *y*-side R-squared (dotted lines refer to PLS-PM)

The fact that some parameters change substantially as a function of $\alpha$ and the extent to which PLS-PM estimates are similar to FPV ones do not tell us much about the consistency of PLS-PM with respect to the formative-reflective scheme. In order to validate the consistency of PLS-PM, we must inspect rotations of exogenous LVs and R-squared values as a function of $\alpha$. Rotations of exogenous LVs, shown in Table 7 and Fig. 4, represent Pearson’s correlations of LV scores with respect to the case $\alpha =0$. We see that ER and SF constructs rotate pretty slight: the correlation between LV scores given $\alpha =0$ and $\alpha =1$ is 0.95 for ER, and 0.991 for SF. Instead, the rotation for CR is higher (0.86 between the case $\alpha =0$ and the case $\alpha =1$) but still moderate according to the examples in Fattore et al. (2018, Sect. 4), especially because the decline of correlation occurs pretty later, i.e., near $\alpha =0.7$. The correlation between PLS-PM and FPV with $\alpha =0$ is above 0.96 for each exogenous LV, suggesting a substantial similarity between the scores extracted by PLS-PM and by the FPV method. Furthermore, R-squared values from the FPV method are unchanged at the first two decimals across all the values of $\alpha$ for both the *x*- and the *y*-side, as shown in Table 8. Also, there is a very small difference with the ones provided by PLS-PM, suggesting that, in this case study, the PLS algorithm has not weakened the fit of the formative part of the model at the expense of the reflective one. For these reasons and because PLS-PM provides a performance-based measure of TDC, in the following, we discuss PLS-PM results, leaving the FPV method as an assessment criterion, as also suggested in Fattore et al. (2018).

**Table 7:** Rotation of exogenous LVs resulting from the FPV method. The values reported are Pearson’s correlations with respect to the case $\alpha =0$

Model	ER	CR	SF
$\alpha =0.0$	1.000	1.000	1.000
$\alpha =0.1$	0.999	0.988	1.000
$\alpha =0.2$	0.998	0.969	1.000
$\alpha =0.3$	0.994	0.950	0.999
$\alpha =0.4$	0.990	0.934	0.998
$\alpha =0.5$	0.984	0.919	0.997
$\alpha =0.6$	0.978	0.904	0.996
$\alpha =0.7$	0.971	0.892	0.995
$\alpha =0.8$	0.963	0.880	0.993
$\alpha =0.9$	0.957	0.870	0.992
$\alpha =1.0$	0.950	0.860	0.991
PLS-PM	0.979	0.960	0.996

**Table 8:** R-squared values resulting from the FPV method. The *y*-side R-squared $R^2_y$ corresponds to the redundancy of the TDC construct, computed as the inner R-squared multiplied by the average variance extracted. The *x*-side R-squared $R^2_x$ is the mean between the R-squared values of the exogenous LVs: $R^2_\text {ER}$, $R^2_\text {CR}$ and $R^2_\text {SF}$

Model	$R^2_x$	$R^2_\text {ER}$	$R^2_\text {CR}$	$R^2_\text {SF}$	$R^2_y\equiv R^2_\text {TDC}$
$\alpha =0.0$	0.419	0.361	0.558	0.446	0.162
$\alpha =0.1$	0.419	0.360	0.558	0.446	0.169
$\alpha =0.2$	0.418	0.359	0.557	0.445	0.172
$\alpha =0.3$	0.418	0.358	0.557	0.444	0.174
$\alpha =0.4$	0.416	0.356	0.557	0.443	0.176
$\alpha =0.5$	0.415	0.355	0.557	0.442	0.177
$\alpha =0.6$	0.413	0.354	0.557	0.441	0.178
$\alpha =0.7$	0.412	0.353	0.556	0.440	0.178
$\alpha =0.8$	0.410	0.352	0.556	0.439	0.179
$\alpha =0.9$	0.408	0.351	0.556	0.438	0.179
$\alpha =1.0$	0.406	0.350	0.555	0.437	0.179
PLS-PM	0.414	0.356	0.557	0.442	0.176

### Discussion

The empirical analysis carried out by PLS-PM and FPV methods confirms the validity of the specified model. The results from the two methodologies basically rend us a similar picture of TDC of Italian municipalities. The structural part of the model shows the endowed resources as the primary driver of TDC. This result agrees with Mazanec et al. (2007) but contrasts with Alves and Nogueira (2015) and, above all, with Conti et al. (2020), who found created resources as the most important determinant of TDC. Even though Conti et al. (2020) used data similar to ours, we have analysed a subset of municipalities. In addition, their results may be affected by the imputation of missing data. In facts, as explained in Conti et al. (2020, p. 1755), missing data on nights spent were imputed proportionally with the number of beds in accommodation facilities, which is an indicator of the supply structure of the destination (i.e., created resources). This imputation may have inflated the association between TDC and created resources.

The weakness of the Sustainability construct agree with other studies (Gooroochurn and Sugiyarto 2005; Conti et al. 2020). The proposed variables (i.e., percentage of waste in separate collection, and absence/presence of protected areas) are likely poor indicators of sustainability, and subjective measures might be more effective (i.e., the perception of environmental status and protection).

**Fig. 5:**
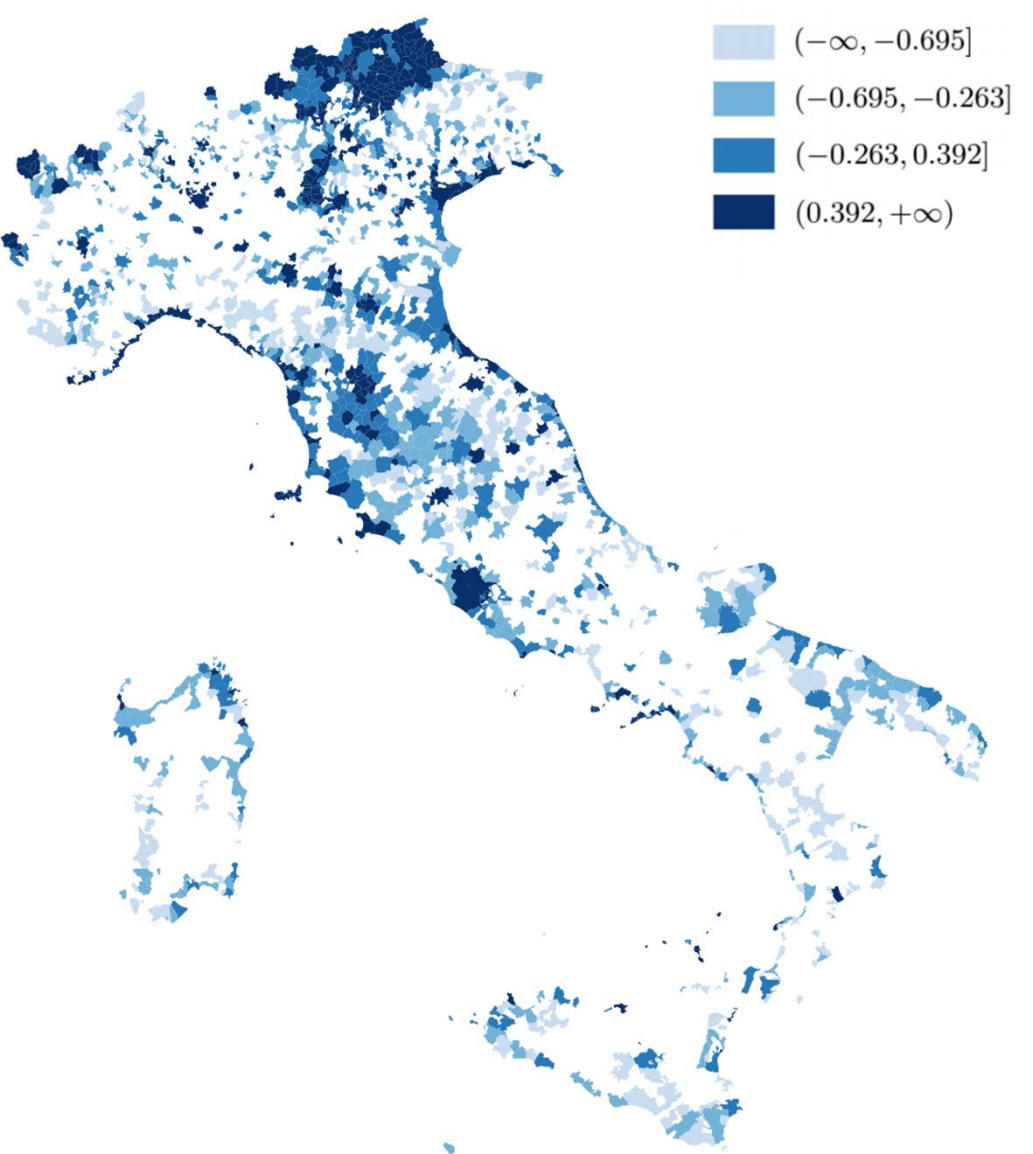
Map of Italian municipalities coloured according to TDC scores (classes based on quintiles) from PLS-PM estimation. Municipalities not included in the study are coloured in white

**Table 9:** TDC scores from PLS-PM estimation: top 20 municipalities and rank of some noteworthy big cities. ‘CHL’: cultural, heritage, landscape

Rank	TDC score	Municipality	Region	Type of locality
1	6.023	Cortina d’Ampezzo	Veneto	Mountain with CHL
2	5.748	Capri	Campania	Coastal with CHL
3	5.723	Lignano Sabbiadoro	Friuli-Venezia Giulia	Coastal
4	5.123	Selva di Val Gardena	Trentino-Alto Adige	Mountain with CHL
5	4.990	Sorrento	Campania	Sea with CHL
6	4.513	Limone sul Garda	Lombardia	Lake
7	4.493	Ortisei	Trentino-Alto Adige	Mountain with CHL
8	4.401	Andalo	Trentino-Alto Adige	Mountain with CHL
9	4.308	Corvara in Badia	Trentino-Alto Adige	Mountain with CHL
10	4.052	Cavallino-Treporti	Veneto	Coastal
11	4.032	Riccione	Emilia-Romagna	Coastal
12	3.981	Alassio	Liguria	Coastal
13	3.977	Positano	Campania	Coastal with CHL
14	3.882	Forte dei Marmi	Toscana	Coastal
15	3.713	Diano Marina	Liguria	Coastal
16	3.709	Milano	Lombardia	Big city
17	3.525	Amalfi	Campania	Coastal with CHL
18	3.517	Canazei	Trentino-Alto Adige	Mountain with CHL
19	3.458	Cattolica	Emilia-Romagna	Coastal
20	3.194	Anacapri	Campania	Coastal with CHL
21	3.153	Roma	Lazio	Big city
23	3.107	Venezia	Veneto	Big city
41	2.641	Firenze	Toscana	Big city
92	1.823	Napoli	Campania	Big city
148	1.336	Bologna	Emilia Romagna	Big city
483	0.210	Palermo	Sicilia	Big city

**Table 10:** Summary of TDC scores from PLS-PM estimation by type of municipality

Category	ER	CR	SF	TDC	%
Big cities	1.806	$-0.080$	3.717	1.658	0.80
Cultural, historical, landscape	$-0.113$	$-0.183$	0.112	$-0.175$	15.3
Coastal	$-0.087$	0.047	0.143	0.063	14.2
Lake	$-0.565$	0.054	0.150	0.198	4.3
Mountain	0.193	0.113	$-0.608$	$-0.137$	11.5
Thermal	$-0.287$	$-0.181$	$-0.190$	$-0.175$	2.1
Coastal with cultural, heritage, landscape	0.622	0.412	0.429	0.440	12.1
Mountain with cultural, heritage, landscape	0.834	0.104	$-0.472$	0.460	9.5
More tourist vocations	$-0.131$	0.377	$-0.058$	0.133	6.5
Others	$-0.518$	$-0.346$	$-0.002$	$-0.346$	23.7

Figure 5 displays the map of Italian municipalities coloured according to TDC scores (classes based on quintiles) from PLS-PM estimation, and Table 9 reports the top 20 destinations based on these scores. The type of municipality has been identified by ISTAT through geographical (i.e., proximity to coasts, mountains, etcetera) and anthropic criteria (infrastructural endowment, population density, etcetera) by including several fine indicators about cultural attractions (e.g., *Borghi più belli*, *Bandiera arancione*, etcetera), and about the impact and the structure of tourism activities (overnight stays, employment in tourism activities, etcetera). Our findings emphasize that big cities with a multifaceted tourism^1^, together with sea and mountain destinations with cultural attractions, are the best ranked ones^2^.

Assuming the soundness of the classification provided by ISTAT, Table 10 may also work as an external criterion to assess the meaning of the extracted LVs. As expected, big cities have higher score of the constructs Endowed Resources (ER, i.e., they are historical cities) and Supporting Factors (SF), while the Created Resources (CR) construct may suffer of differences in the size of resident population, although we took into account this problem at least for the indicator $X_\text {CR,1}$. Also, we note the reasonably lower score of the Supporting Factors (SF) construct for mountain destinations. In all, the extracted LVs effectively discriminate between mountain and coastal areas on one hand, and between mountain and coastal areas augmented with cultural and heritage resources on the other hand. The same can be said for the ‘Others’ category, which includes municipalities without a definite specialization in tourism. Moreover, the results indicate a weak TDC of lake and thermal destinations. In Italy, the thermal sector is heterogeneous and fragmented, and typically unable to exploit relevant built heritage (Faroldi et al. 2019).

An evident inconsistency in the results relies in the average LV scores for the category ‘Cultural and historical destinations’, in particular the score of the Endowed Resources (ER) construct, which is negative (i.e., below the overall mean) instead of positive (i.e., above the overall mean) as expected. Besides this weakness, the formative-reflective specification can be considered appropriate in this case study, as proved by the model assessment performed in Sect. 4.3.1 and 4.3.2.

## Concluding remarks

This article has proposed an operationalization of Tourism Destination Competitiveness (TDC) for a large number of destinations under the formative-reflective model, its validity has been assessed through a novel methodology, which also appears as a valuable alternative to PLS-PM.

On a conceptual basis, a limitation of our model relies on the absence of a construct representing demand conditions (Dwyer and Kim 2003, p. 398), i.e., those factors concerned with demand-awareness, perception and preferences (pull factors), which require subjective data. Anyway, the indicators employed in our study are in line with the other several applications of PLS-PM to TDC of countries, regions or municipalities, where secondary objective data are exclusively employed.

On an empirical basis, although there is a weakness in the representation of the multifaceted TDC determinants of Italian municipalities (especially for endowed resources), our study supports the appropriateness of the formative-reflective scheme for the assessment of TDC. The benefit of a complex theoretical framework of TDC may effectively emerge when it is translated into an operationalized cause-effect logic (Mazanec et al. 2007). Our results encourage us to improve the analysis by including more indicators, possibly adopting those used by ISTAT in the categorization of tourist municipalities.

TDC scores extracted by PLS-PM and FPV in the formative-reflective model are different in some extent: PLS-PM is intended to derive a performance-based measure of TDC, whereas FPV returns a linear combination of the exogenous constructs (i.e., TDC determinants). This discrepancy is inherent in the formative-reflective scheme, because different conceptual interpretations are possible (Diamantopoulos et al. 2008; Jarvis et al. 2003): on one hand, a higher-order composite that impacts on MVs, or, on the other hand, a reflective endogenous construct influenced by the exogenous LVs through the structural part of the model, like in our study.

## Footnotes

1 The ‘Big cities’ category comprises twelve cities, including the top historical and cultural destinations of Italy: Torino, Genova, Milano, Verona, Venezia, Bologna, Firenze, Roma, Napoli, Bari, Palermo, Catania

2 Law n. 77 (July, 17, 2020), comprising urgent interventions related to the epidemiological emergency from COVID-19, provides a classification of economic activities (defined by ISTAT) with reference to areas with high tourist density, in order to target support measures in favour of businesses in the trade sectors, catering and accommodation facilities
